# Sulfur-Induced Lattice Disordering in Halide Electrolytes Enables Superionic Sodium-Ion Conduction

**DOI:** 10.1021/acsnano.6c11189

**Published:** 2026-10-29

**Authors:** Feng Jin, Bingxu Wang, Meenal Agrawal, Ingeborg Sellæg Ellingsen, Guobiao Jin, Lulu Alluhaibi, Alexey Maximenko, Marcin Zając, Dragos Stoian, Kenneth Marshall, Wouter Van Beek, Feng Pan, Daniel Rettenwander

**Affiliations:** † School of Advanced Materials, 429362 Peking University Shenzhen Graduate School, Shenzhen 518055, China; ‡ Department of Material Science and Engineering, NTNU Norwegian University of Science and Technology, Trondheim 7491, Norway; § National Synchrotron Radiation Centre SOLARIS, 37799 Jagiellonian University, Czerwone Maki 98, 30-392 Kraków, Poland; ∥ Swiss-Norwegian Beamlines, European Synchrotron Radiation Facility, 71 Ave. des Martyrs, Grenoble 38000, France; ⊥ Christian Doppler Laboratory for Solid-State Batteries, NTNU Norwegian University of Science and Technology, Trondheim 7491, Norway; # AIT Austrian Institute of Technology GmbH, Giefinggasse 2, Vienna 1210, Austria; ∇ Institute of Chemical Technologies and Analytics, Vienna University of Technology, Getreidemarkt 9, Vienna 1060, Austria

**Keywords:** Amorphous solid electrolytes, Sodium-ion conduction, Mixed-anion chemistry, Local coordination structure, All-solid-state sodium batteries

## Abstract

Amorphous solid electrolytes provide a promising route to overcoming the ion-transport limitations of crystalline halide frameworks, yet achieving superionic Na^+^ conduction remains challenging. Herein, we report a mixed-anion amorphous Na-ion solid-state electrolyte, TaCl_5_-0.5Na_2_S (NTSC), synthesized via high-energy ball-milling, which exhibits an ultrahigh room-temperature ionic conductivity of 2.9 mS/cm and a low activation energy of 0.276 eV. Structural analyses reveal a highly disordered framework composed of Ta-centered bipolyhedra interconnected via corner-sharing sulfur atoms [Ta_2_Cl_10_S], [TaCl_5_S], [TaCl_4_S], and [TaCl_4_S_2_], evidenced by comprehensive characterizations, forming a flexible, disordered amorphous network. Ab initio molecular dynamics (AIMD) simulations identify continuous Na^+^-diffusion pathways with low migration barriers. Complementary topological analyses further indicate that sulfur incorporation leads to local lattice distortions, shortens Na–Na hopping distances, and enhances framework flexibility, collectively enabling fast, multidimensional Na^+^ transport. When applied in all-solid-state sodium-ion batteries (ASSNIBs), NTSC enables high reversible capacity (111.95 mAh/g with a Coulombic efficiency of 92.6% at 0.1 C) and excellent cycling stability (76.9% initial capacity retention after 200 cycles at 0.2 C) at room temperature. This work establishes mixed-anion amorphization as an effective design strategy for high-performance Na-ion solid electrolytes.

## Graphical abstract

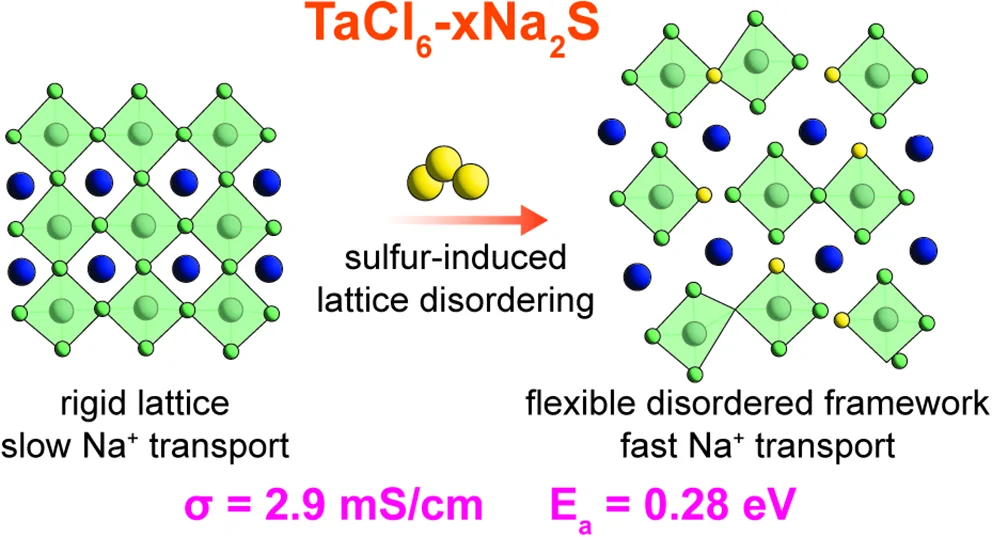

## Introduction

All-solid-state sodium-ion batteries (ASSNIBs) have emerged as promising candidates for grid-scale energy storage and cost-sensitive electric mobility applications, owing to the natural abundance, low cost, and environmentally benign nature of sodium, together with the intrinsic safety advantages afforded by solid electrolytes.^1^ A critical challenge in realizing high-performance ASSNIBs is the development of Na-ion solid-state electrolytes (Na-SSEs) that simultaneously deliver high ionic conductivity (>0.1 mS/cm at room temperature), negligible electronic conductivity, and robust electrochemical, chemical, and mechanical compatibility with both cathode and anode materials.^2^

Over the past decades, a wide range of Na-SSEs has been explored, including oxide-based NASICON-type conductors (Na_1+*x*_Zr_2_Si_*x*_P_3–_ *_x_* O_12_), sulfide electrolytes (e.g., Na_3_PS_4_ and Na_3_SbS_4_), and halide-based systems (e.g., Na_3_YCl_6_ and Na_2_ZrCl_6_).^3,4^ Among these material classes, chloride-based Na-SSEs have attracted increasing attention due to their wide electrochemical stability windows and favorable mechanical properties and processability.^5,6^ However, their practical deployment remains constrained by intrinsically limited Na^+^ conductivity, which originates from close-packed halide lattices, rigid anion frameworks, and fixed Na^+^ coordination environments. To mitigate these limitations, aliovalent cation doping strategies—such as substitution with Er^3+^, Hf^4+^, and other rare-earth cations—have been employed to introduce Na^+^ vacancies and lattice disorder, yielding conductivities exceeding 0.1 mS/cm at room temperature.^7,8^ Despite these advances, further enhancement of Na^+^ transport is essential for competitive ASSNIB performance.

In parallel, amorphous solid electrolytes have gained increasing interest as alternatives to crystalline counterparts, as the absence of long-range order eliminates resistive grain boundaries and relaxes crystallographic constraints on ion migration. The resulting structural and energetic disorder enables isotropic Na^+^ transport through a heterogeneous energy landscape and allows for dynamic local structural relaxation during ion hopping. To date, extensive efforts have focused on oxide-based glassy Na-SSEs (e.g., Na_2_O–P_2_O_5_, Na_2_O–B_2_O_3_, and Na_2_O-SiO_2_) and sulfide-based systems (e.g., Na_2_S–P_2_S_5_–SiS_2_, Na_3_PS_4_–Na_4_GeS_4_, Na_2_S–P_2_S_5_, and Na_2_S–B_2_S_3_).^9–11^ In these materials, Na^+^ diffusion occurs through disordered anion networks composed of motifs such as S^2–^, PS_4_^3–^, and P_2_S_7_^4–^ units. Nevertheless, strong Coulombic interactions, limited framework flexibility, and nonideal Na^+^ coordination environments often lead to insufficient room-temperature ionic conductivities, thereby hindering their widespread implementation in ASSNIB architectures.

More recently, amorphous chloride-based Na-SSEs have emerged as a compelling new material platform, exhibiting room-temperature ionic conductivities exceeding 1 mS/cm. Representative systems, including NaTaCl_6_-derived glasses and mixed-anion compositions such as Na_2_O_2_–TaCl_5_ and Na_2_CO_3_–TaCl_5_, demonstrate that the incorporation of multiple anionic species fundamentally reshapes the local structural and electrostatic landscape.^12–15^ Mixed-anion frameworks introduce pronounced structural disorder, weaken Na^+^-anion interactions, and generate a broad distribution of Na^+^ coordination environments, collectively flattening the diffusion energy landscape and enabling rapid ion transport.^13^ These advances highlight the critical role of anion chemistry and amorphization in overcoming the intrinsic conductivity limitations of crystalline halide electrolytes and opening new avenues for the rational design of high-performance Na-SSEs.

Herein, we report a new amorphous superionic Na-SSE, TaCl_5_-0.5Na_2_S (NTSC), synthesized via high-energy ball-milling using TaCl_5_ and Na_2_S as precursors. The resulting amorphous NTSC matrix consists primarily of Ta-centered bipolyhedra interconnected via corner-sharing sulfur atoms [TaCl_5_S], [TaCl_4_S], and [TaCl_4_S_2_]. This distinctive mixed-anion local structure weakens Na^+^-lattice interactions and promotes a flexible, disordered framework, facilitating rapid long-range Na^+^ transport and yielding a high ionic conductivity of 2.9 mS/cm at 25 °C. Sulfur incorporation induces local lattice distortions, shortens Na–Na hopping distances, and enhances framework flexibility, which collectively enable fast, multidimensional Na^+^ transport. When paired with an uncoated NaCrO_2_ cathode, NTSC-based ASSNIBs deliver a high initial discharge capacity of 111.95 mAh/g with a Coulombic efficiency of 92.6% and retain 76.9% of their capacity after 200 cycles at 0.2 C and 25 °C. This work introduces a new class of amorphous mixed-anion Na-SSEs, demonstrates their effectiveness in enabling high-performance ASSNIBs, and provides design principles for the future development of amorphous halide electrolytes.

## Results and Discussion

A series of amorphous solid electrolytes with nominal compositions TaCl_5_-*x*Na_2_S (*x* = 0.25, 0.5, 0.75, and 1.0), namely, TaCl_5_-*x*Na_2_S, were synthesized via high-energy ball-milling using TaCl_5_ and Na_2_S as precursors (Figure 1a).

**Figure 1:**
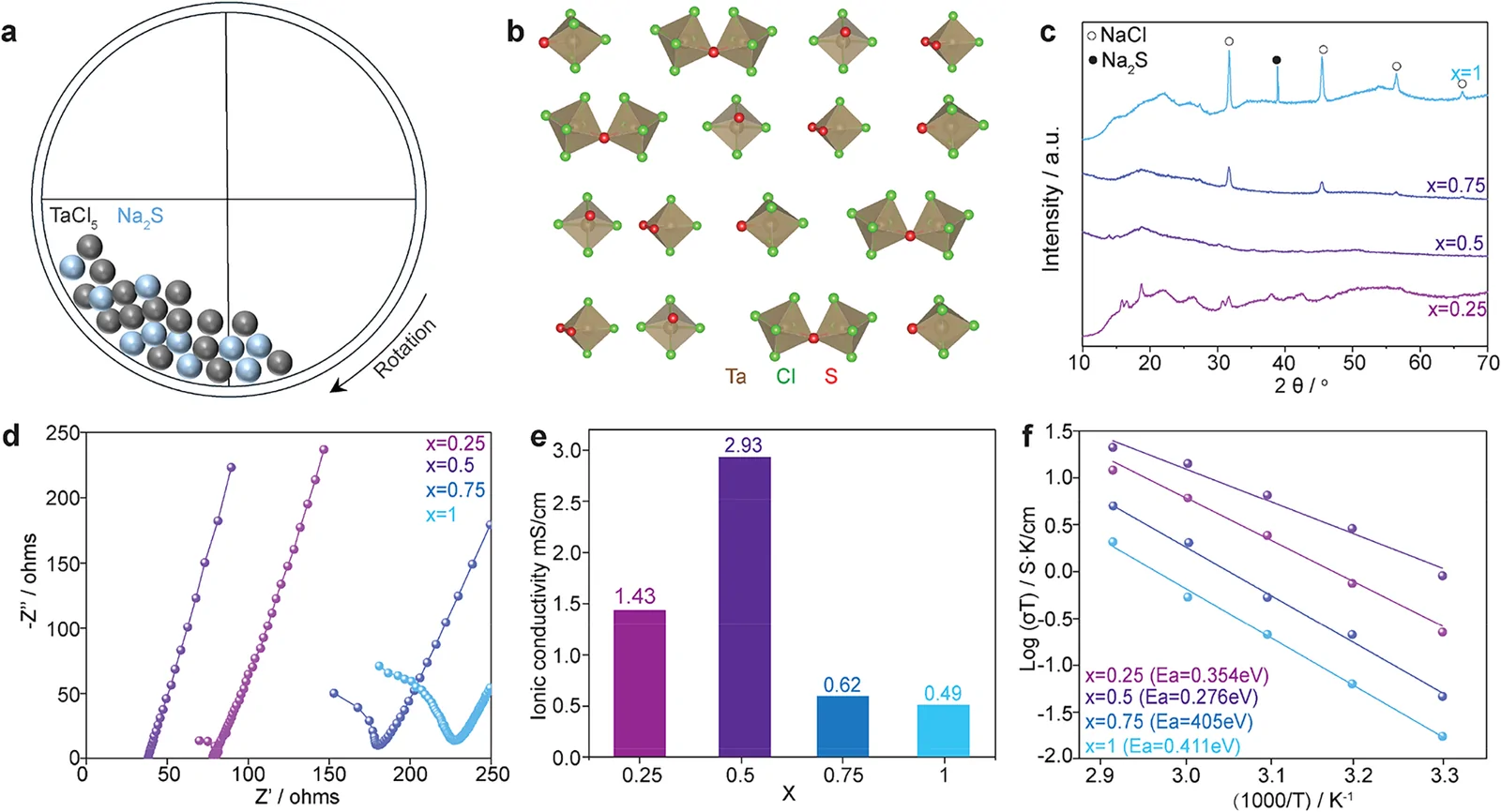
Synthesis and ionic transport properties of amorphous TaCl_5_-*x*Na_2_S solid electrolytes. (a) Schematic illustration of the high-energy ball-milling synthesis of TaCl_5_-*x*Na_2_S using TaCl_5_ and Na_2_S as precursors. (b) Structural model illustrating the local mixed-anion coordination environment in amorphous TaCl_5_-0.5Na_2_S. (c) X-ray diffraction (XRD) patterns of TaCl_5_-*x*Na_2_S (*x* = 0.25–1.0), highlighting the formation of a fully amorphous phase at *x* = 0.5. (d) Nyquist plots of TaCl_5_-*x*Na_2_S (*x* = 0.25–1.0) SSEs and the corresponding calculated Na^+^ ionic conductivities (e) at room temperature as a function of Na_2_S content, identifying TaCl_5_-0.5Na_2_S as the optimal composition. (f) Arrhenius plots of Na^+^ conductivity for TaCl_5_-*x*Na_2_S SSEs and their calculated values of activation energy.

The degree of amorphization was evaluated by X-ray diffraction (XRD), as the absence of sharp Bragg reflections is indicative of a glassy or amorphous structure. The corresponding XRD patterns are shown in Figure 1c and reveal that a fully amorphous phase is obtained at *x* = 0.5 (see also Figure S2). In contrast, samples with higher Na_2_S content (x = 0.75 and 1.0) exhibit the emergence of distinct diffraction peaks, which can be unambiguously assigned to crystalline NaCl and residual Na_2_S phases, indicating phase separation and incomplete amorphization at elevated *x* values.^13,16^

Accordingly, the thermal properties of the optimized TaCl_5_-0.5Na_2_S composition (denoted as NTSC) were examined by differential scanning calorimetry (DSC). The DSC trace (Figure S4) exhibited a clear glass transition at 193 °C without any detectable crystallization exotherms upon heating, indicating a stable glass framework with moderate network rigidity, which is favorable for maintaining dynamic local environments that support Na^+^ migration.

The Na^+^ conductivities of the TaCl_5_-*x*Na_2_S series were evaluated by electrochemical impedance spectroscopy (EIS) over a temperature range of 25–70 °C. Representative Nyquist plots of NTSC display a depressed semicircle in the high-frequency region, corresponding to bulk ionic transport, followed by a low-frequency spike associated with ion-blocking electrodes (Figures 1d and S5). Based on EIS fitting (details in Experimental Section), the optimized amorphous NTSC electrolyte delivered a room-temperature ionic conductivity of up to 2.9 mS/cm at 25 °C (Figure 1e). The Arrhenius behavior of the Na-ion conductivity for all compositions (Figure 1f) in the temperature range between 25 and 70 °C has been studied for all compositions. The corresponding activation energy is as low as 0.276 eV, highlighting its exceptionally favorable ion-transport kinetics. To benchmark its performance, the room-temperature ionic conductivity of NTSC is compared with that of representative inorganic Na-SSEs reported in the literature. As summarized in Table S1, NTSC outperforms most reported Na-SSEs, while some recently reported mixed-anion systems (e.g., NTOC) exhibit comparable ionic conductivities (4.62 mS/cm) and activation energy (0.30 eV).^13^

To elucidate the structural features of amorphous NTSC, a combination of complementary characterization techniques sensitive to short-range atomic order was employed, including Raman spectroscopy, X-ray photoelectron spectroscopy (XPS), X-ray absorption spectroscopy (XAS), and X-ray pair distribution function (xPDF) analysis. Given its superior ionic conductivity and representative structural features, detailed characterization was focused on the optimized TaCl_5_-0.5Na_2_S composition.

Raman spectroscopy provides direct insight into the local bonding environments and coordination motifs in amorphous materials. The Raman spectrum (Figure 2a) of NTSC exhibits a broad, featureless profile compared to the sharp and well-defined vibrational modes of the crystalline TaCl_5_ and Na_2_S precursors, consistent with the formation of a highly disordered amorphous structure. The characteristic vibrational signatures of TaCl_5_ and Na_2_S are largely suppressed or significantly broadened in NTSC, indicating a substantial disruption of long-range periodicity. Notably, broad bands appearing in the 150–350 and 300–400 cm^–1^ regions can be attributed to Ta-centered polyhedral vibrations coupled with sulfur coordination, suggesting the formation of corner-sharing [Ta_2_Cl_10_S] oligomeric units within the amorphous matrix.^17,18^ Sulfur bridging is further evidenced by the Ta 4*f* X-ray photoelectron spectroscopy (XPS) spectrum with extra peaks located at around 27 and 29 eV (Figure 2b).

**Figure 2:**
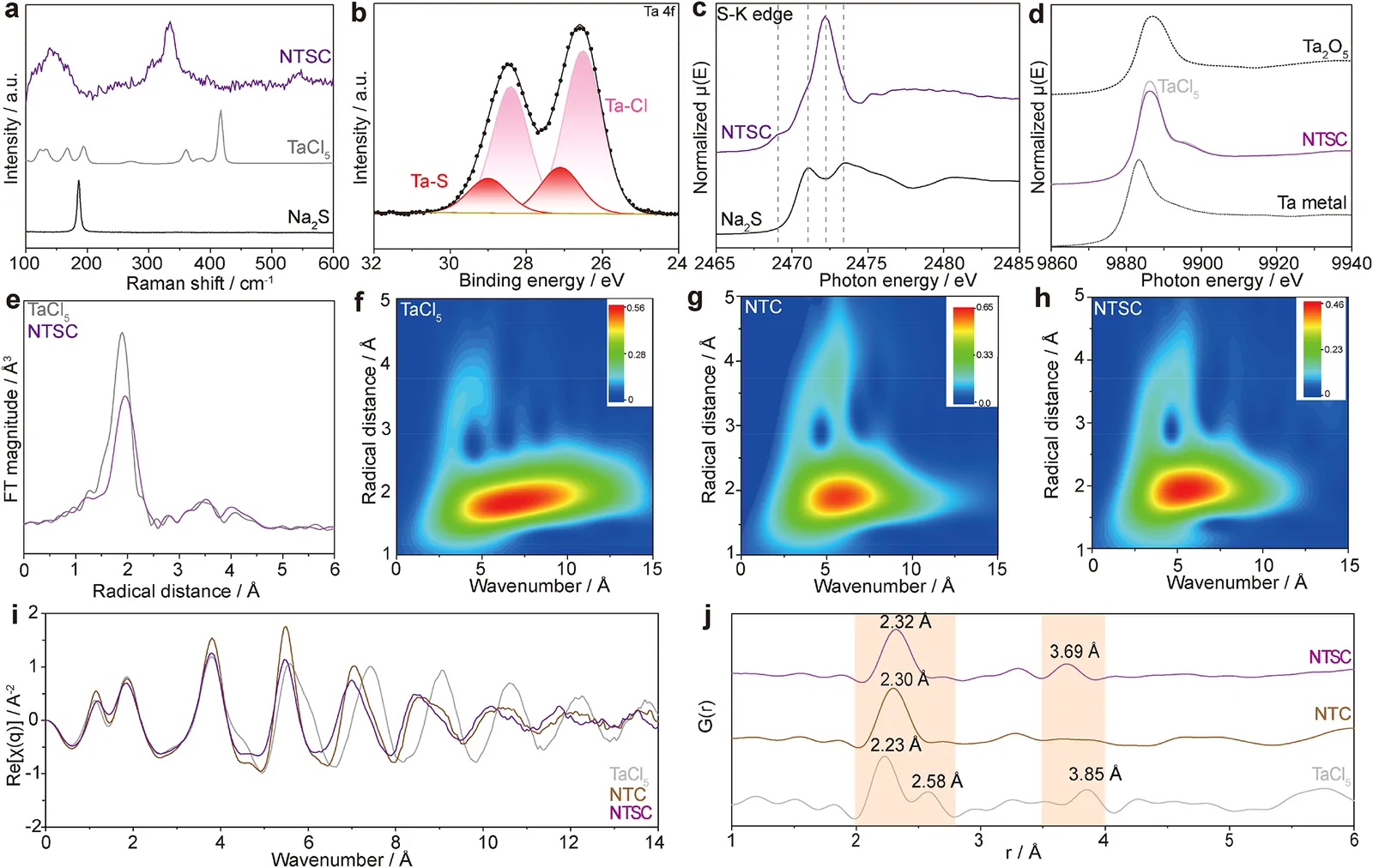
Structural characterization of the amorphous NTSC electrolyte. (a) Raman spectra of TaCl_5_, Na_2_S, and NTSC, showing the suppression and broadening of precursor vibrational modes upon amorphization. (b) Ta 4*f* X-ray photoelectron spectroscopy (XPS) spectrum of NTSC, revealing mixed sulfur coordination environments. (c) Normalized S *K*-edge X-ray absorption near-edge structure (XANES) spectra of Na_2_S and NTSC, indicating preserved sulfur oxidation state and a largely unchanged Na^+^ local chemical environment in the amorphous framework. (d) Normalized Ta *L*_3_-edge X-ray absorption near-edge structure (XANES) spectra of TaCl_5_ and NTSC. (e) Fourier-transformed (FT) curves at R space of TaCl_5_ and NTSC. Wavelet-transformed extended X-ray absorption fine structure (WT-EXAFS) at the Ta *L*_3_-edge for TaCl_5_ (f), NTC (g), and NTSC (h), resolving nearest-neighbor Ta–S and Ta–Cl interactions and medium-range Ta–Ta correlations. (i) Ta *L*_3_-edge *k* ^2^-weighted EXAFS spectra, *k* ^2^χ­(*k*), of TaCl_5_, NTC and NTSC. (j) X-ray pair distribution function (xPDF) profiles of TaCl_5_, NaTaCl_6_ (NTC), and NTSC, highlighting the emergence of pronounced Ta–Ta correlations in NTSC consistent with oligomeric Ta-centered frameworks interconnected via corner-sharing sulfur atoms.

Complementary to Raman spectroscopy and XPS, which are sensitive to local vibrational environments and chemical bonding, X-ray absorption spectroscopy (XAS) offers element-specific information on local coordination geometry and electronic structure. The normalized S *K*-edge X-ray absorption near-edge structure (XANES) spectrum of NTSC exhibits two shoulder features at around 2471.1 and 2473.4 eV, coinciding with those of Na_2_S, indicating a similar sulfur oxidation state (Figure 2c).^19^ In addition, two distinct features at around 2469.0 and 2472.2 eV are observed, which can be attributed to Ta–S interactions, suggesting that the S 1*s* → *p/d* hybridized transitions in NTSC are influenced by coordination with Ta^5+^ rather than solely by Na^+^. The Na K-edge XANES spectra of NTSC and Na_2_S (Figure S6) show close correspondence in both peak positions and line shapes, indicating that the local chemical environment of Na^+^ remains largely unchanged upon amorphization, consistent with its role as a weakly coordinated, mobile charge carrier. Similarly, the Cl *K*-edge XANES spectrum of NTSC exhibits no pronounced peak shifts or line-shape changes relative to the TaCl_5_ precursor (Figure S7), suggesting that Cl retains a comparable local coordination environment in the amorphous state.

Furthermore, the Ta *L*_3_-edge XANES and corresponding Fourier transform-EXAFS reveal systematic modifications of the local electronic and structural environment upon S incorporation into the TaCl_5_-derived material. In TaCl_5_, Ta^5+^ (5d^0^) is coordinated by six Cl atoms forming distorted edge-sharing TaCl_6_ octahedra, with Ta–Cl bond lengths ranging from 2.26 to 2.56 Å. The XANES spectrum of such a distorted octahedral structure shows a broad peak with an indistinct top at the white-line position. Moreover, the structural distortion of the octahedral symmetry generally leads to a small splitting of the 5d states, as can be precisely seen in the second derivative plot displaying two minima (ca. 2.8 eV split in TaCl_5_) (Figure S8). Upon S incorporation, white-line intensity of NTSC decreases without a noticeable edge shift, indicating that Ta largely retains its pristine +5 oxidation state while exhibiting reduced unoccupied 5d density of states, consistent with enhanced metal–ligand covalency due to the more covalent nature of S relative to Cl. The postedge region (∼9890 to 9900 eV) becomes slightly damp, suggesting increased structural disorder (Figure 2d). The second-derivative spectrum shows broadened features and a modest increase in apparent splitting (∼0.4 eV), indicative of mixed Ta–Cl/S coordination environments (Figure S8). Fourier transform-EXAFS analysis shows that the first-shell peak shifts to higher radial distance (from 1.89 Å in TaCl_5_ to 1.96 Å in NTSC, uncorrected for phase shift), accompanied by peak broadening and reduced amplitude, reflecting an increase in average Ta–ligand bond length and greater structural heterogeneity, consistent with partial substitution of Cl by larger, less electronegative S atoms (typical Ta–S ≈ 2.47 Å, obtained from crystallographic databases) (Figure 2e). The observed broadening and amplitude reduction of the first-shell peak of the sample suggests a distribution of local environments arising from mixed Ta–Cl and Ta–S coordination and increased disorder. Wavelet transform-EXAFS further supports increased disorder, as evidenced by faster *k*-space damping at high *k* for NTSC. In contrast, the higher-R region (3–5 Å) remains largely preserved (Figure 2f–2h). Besides, the reduced and broadened first-shell FT contribution, its different apparent R-space position, the more rapid damping of the high-*k* EXAFS oscillations, and the intermediate/weak 3–5 Å correlations demonstrate that NTSC remains structurally distinct from NTC and possesses substantially greater local and medium-range disorder. Together, these results support a scenario in which S incorporation induces local structural and electronic perturbations—manifested as elongated and more heterogeneous Ta–ligand bonding—while maintaining the overall TaCl_5_-derived structural motif.

While EXAFS primarily probes the local coordination environment around a selected absorber, xPDF analysis provides real-space information on both short- and medium-range atomic correlations, independent of long-range periodicity, making it particularly effective for probing amorphous structures. The xPDF of TaCl_5_, NaTaCl_6_ (NTC), and NTSC reveals that in the first coordination shell, two distinct Ta–Cl peaks at 2.23 and 2.58 Å merge in TaCl_5_, while in NTC the bridging Ta–Cl feature disappears due to isolated [TaCl_6_] octahedra, with the main Ta–Cl peak shifting to 2.30 Å. Partial substitution of Cl by S further shifts this peak to 2.32 Å, reflecting the longer Ta–S bond lengths relative to Ta–Cl. In the second coordination shell, the Ta–Ta peak at 3.85 Å in TaCl_5_ vanishes in NTC, consistent with octahedral isolation, but reappears at 3.69 Å in NTSC, arising from corner-sharing [Ta_2_Cl_10_S] octahedra (Figure 2j). These results demonstrate how local and medium-range structural motifs evolve upon Cl substitution and octahedral connectivity changes, highlighting the sensitivity of xPDF to both bond-length variations and coordination topology.^13^

Since S and Cl are neighboring elements with closely related EXAFS backscattering amplitudes and phases and overlapping plausible Ta-X distances, Ta *L*_3_-edge EXAFS alone cannot uniquely partition the first coordination shell into quantitative Cl and S populations. The XAS results should therefore be interpreted as evidence for a strongly reorganized and heterogeneous average Ta-centered environment, while assignment of mixed Ta–Cl/S coordination derives from the convergence of XAS with xPDF. To elucidate the local incorporation of sulfur into the Ta–Cl framework, TOF-SIMS was employed to probe the spatial distribution of characteristic Ta–S-containing fragments. As shown in Figure 3a–3d, multiple Ta–S-related species, including [Ta_2_S^+^], [TaS^–^], [TaS_2_^–^], and [TaClS^–^], are detected throughout the analyzed region, which might relate to motifs like [Ta_2_Cl_10_S], [TaCl_5_S], [TaCl_4_S], and [TaCl_4_S_2_] (Figure 3e). The simultaneous observation of Ta, Cl, and S-containing fragments provides strong evidence that sulfur is incorporated into the Ta–Cl-based network rather than existing solely as an isolated sulfide species. More importantly, the coexistence of multiple Ta–Cl/S fragments suggests that sulfur incorporation does not generate a single uniform coordination environment. Instead, S substitution induces a distribution of locally distinct Ta-centered configurations, ranging from TaCl_5_S- and TaCl_4_S-like units to more S-rich environments such as TaCl_4_S_2_. The corresponding structural models illustrate these possible local coordination motifs and highlight the structural diversity introduced by S incorporation. Collectively, the TOF-SIMS spatial mapping and fragment analysis support the formation of a chemically heterogeneous Ta–Cl/S mixed-anion network with substantial short-range structural disorder.

**Figure 3:**
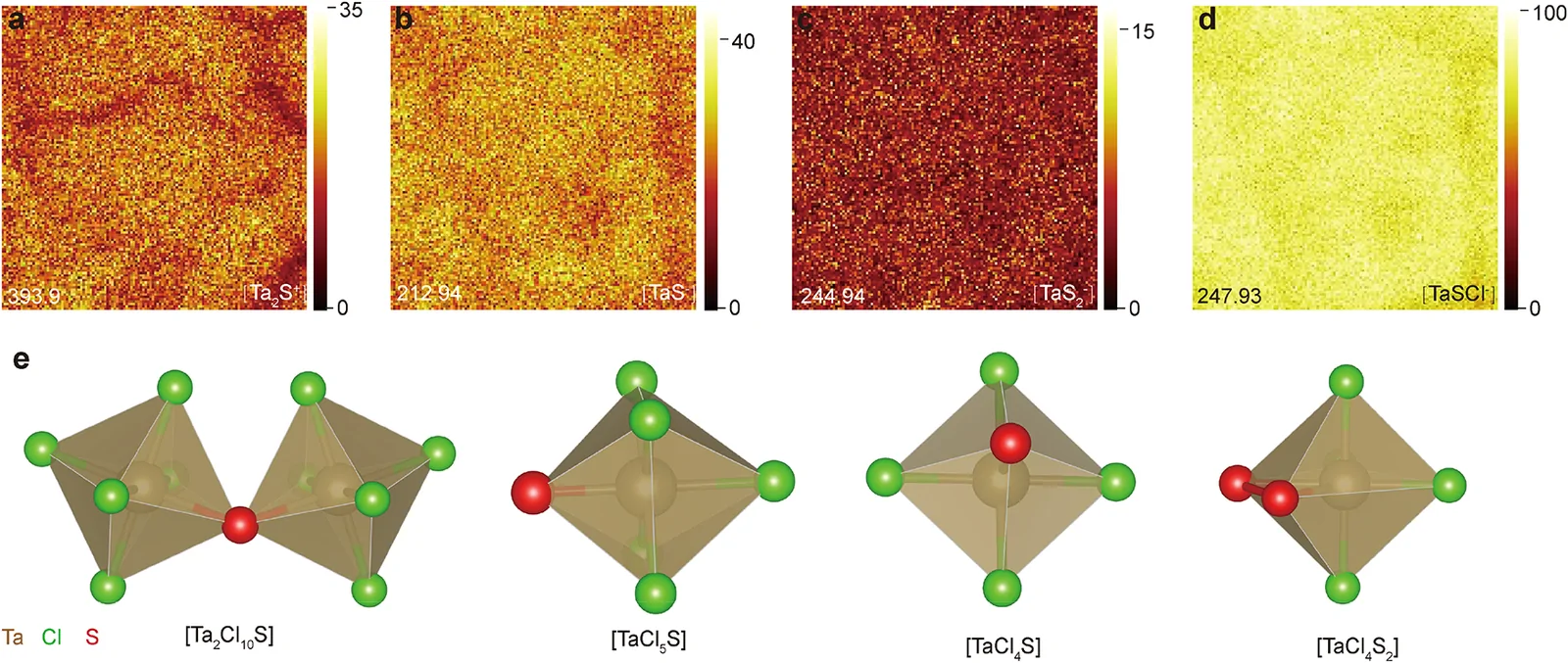
Local structural disorder in the Ta–Cl/S framework. (a–d) Two-dimensional spatial distributions of the characteristic fragments assigned to Ta–S-containing species, including [Ta_2_Cl_10_S], [TaCl_5_S], [TaCl_4_S], and [TaCl_4_S_2_], respectively. (e) Representative structural models of the corresponding Ta–Cl–S coordination units, illustrating the incorporation of S into the Ta–Cl framework and the formation of diverse mixed-anion Ta–Cl/S local environments.

To account for the pronounced structural disorder and diverse local coordination environments revealed by the experimental characterization, an amorphous NTSC structural model was constructed using a melt–quench *ab initio* molecular dynamics (AIMD) procedure (Figure S9a).^20^ The resulting structure exhibits substantial spatial heterogeneity, with Na, S, and Cl preferentially distributed within interconnected regions while Ta-rich regions form a comparatively rigid framework. Such structural heterogeneity provides a plausible structural basis for Na^+^ transport by generating interconnected Na-accessible environments within an amorphous framework. The resulting amorphous structure was therefore used for subsequent Na-ion transport calculations.^21^

Using this amorphous structural model, we performed extended AIMD simulations at 600, 800, 1000, and 1200 K, with each trajectory propagated for 30 ps. The Na^+^ mean-squared displacement (MSD) was calculated from the resulting trajectories, and the initial approximately 2 ps transient regime was excluded from the diffusion analysis. As shown in Figure S10, the subsequent MSD at 800 K exhibits a well-defined linear regime with *R* ^2^ = 0.9701, supporting the determination of the Na^+^ diffusion coefficient from the Einstein relation.^22^ The temperature-dependent diffusion coefficients obtained from the extended AIMD simulations are 7.07 × 10^–6^, 1.56 × 10^–5^, 2.89 × 10^–5^, and 5.22 × 10^–5^ cm^2^ s^–1^ at 600, 800, 1000, and 1200 K, respectively. These values show a systematic increase in Na^+^ mobility with increasing temperature.

To evaluate the statistical reliability of the diffusion coefficients, the 30 ps trajectories were further divided into three temporal intervals, and the diffusion behavior was independently evaluated within these intervals. The resulting variations were used to estimate the uncertainty of the diffusion coefficients, and the corresponding error bars are included in the Arrhenius analysis in Figure S9b. The estimated uncertainties are 1.27 × 10^–6^, 2.38 × 10^–6^, 4.29 × 10^–6^, and 6.63 × 10^–6^ cm^2^ s^–1^ at 600, 800, 1000, and 1200 K, respectively. The resulting Arrhenius relationship gives an apparent activation energy of 0.202 eV, indicating thermally activated Na^+^ transport with a relatively low effective energy barrier.^23^

Extrapolation of the Arrhenius relationship to 300 K gives a Na^+^ diffusion coefficient of 1.33 × 10^–7^ cm^2^ s^–1^. Using the Nernst–Einstein relation, the corresponding ionic conductivity is estimated to be 3.18 mS cm^–1^ at 300 K. This value is close to the experimentally measured room-temperature ionic conductivity of 2.9 mS cm^–1^, with a difference of approximately 9.6%. The calculated conductivity should nevertheless be considered an AIMD-based estimate rather than an exact prediction of the experimental value. In particular, the Nernst–Einstein treatment assumes independent ionic motion and does not explicitly account for collective Na^+^–Na^+^ correlations. The calculation also involves extrapolation from elevated-temperature AIMD simulations to 300 K, while finite simulation time, finite simulation-cell size, and differences between the computational amorphous model and the experimentally synthesized material may further contribute to the discrepancy. Despite these limitations, the close correspondence between the calculated and experimental conductivities supports the consistency of the simulated Na^+^ transport behavior with the experimentally observed high ionic conductivity. The experimentally determined activation energy of 0.276 eV is somewhat higher than the AIMD-derived apparent value of 0.202 eV, but both values indicate a relatively low barrier for thermally activated Na^+^ transport.

While conventional structural analyses mainly focus on identifying dominant migration pathways, they often provide limited information regarding the higher-dimensional connectivity and spatial organization of disordered frameworks. To quantitatively evaluate how the amorphous framework topology influences Na^+^ transport, persistent homology-based topological data analysis was employed to characterize both the Na^+^ subnetwork and the non-Na framework in NTSC and the reference structure.^24^

As shown in Figure 4e,4f, the persistent homology analysis reveals distinct topological characteristics between NTSC and the reference structure.^25^ For the Na^+^ subnetwork, the H_0_ persistence features describe the characteristic spatial organization of Na environments and the distribution of Na-associated connectivity length scales. Compared with the reference structure, NTSC exhibits an increased total H_0_ persistence from 168.82 to 188.05 Å and an increased mean H_0_ persistence from 4.82 to 5.37 Å, indicating a rearranged Na spatial distribution with a larger characteristic length scale induced by the mixed-anion amorphous framework. This reorganization is consistent with the shortened Na–Na hopping distances observed in NTSC, which provide more favorable geometric configurations for Na-ion migration.

**Figure 4:**
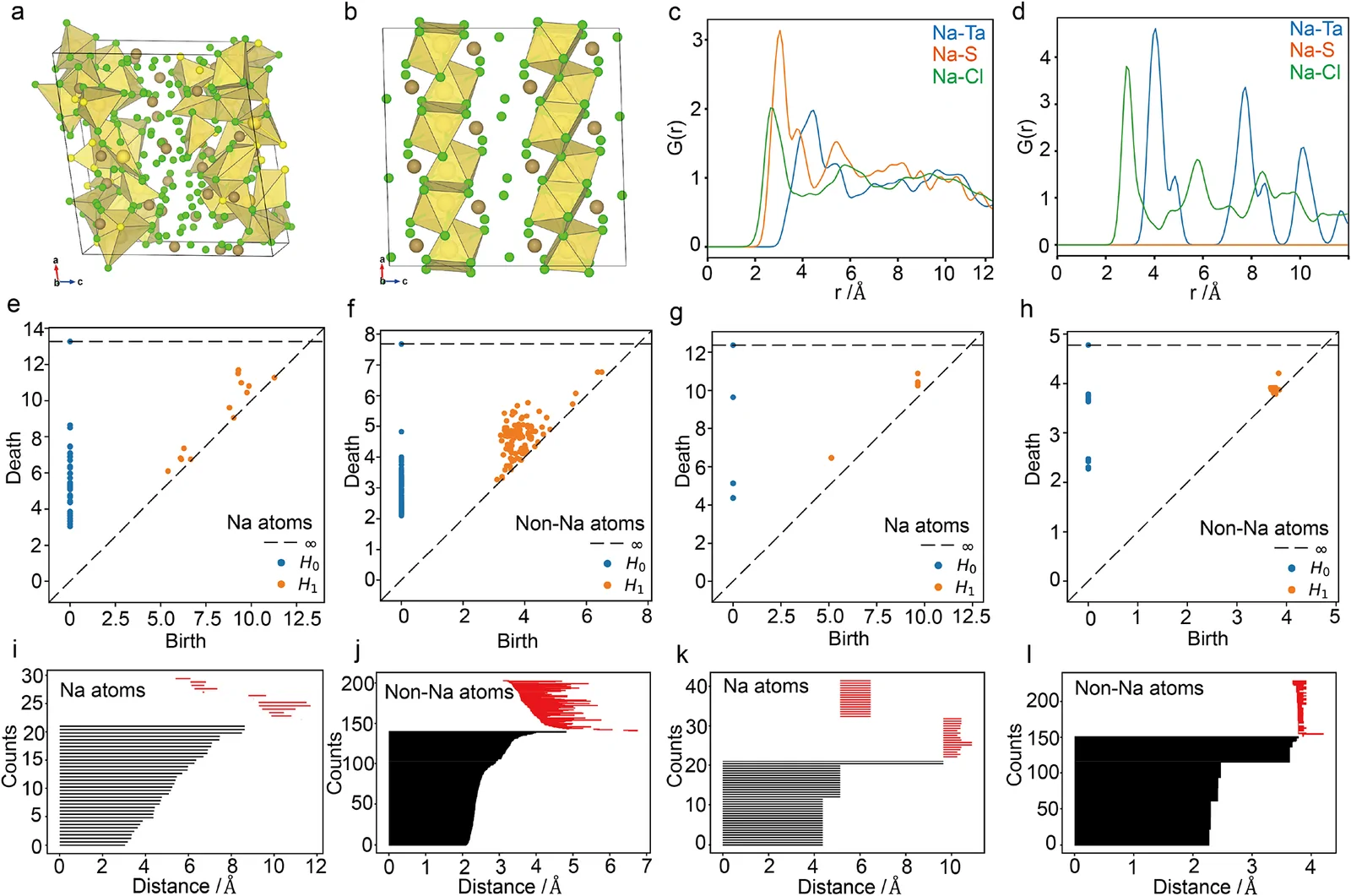
Atomistic origin of fast Na^+^transport in NTSC revealed by simulations and topological analysis. Representative structural models of amorphous NTSC (a) and crystalline NTC (b), with highlighted pathways indicating potential Na^+^ migration channels. (c, d) Radial distribution functions (RDFs) of NTSC (c) and NTC (d), showing shortened Na–Na distances and modified Na-anion correlations in NTSC arising from mixed-anion coordination. Persistent homology analysis of NTSC (e, f) and NTC (g, h), shown separately for the Na-ion subnetwork and the non-Na framework, illustrating differences in Na–Na hopping distances and framework connectivity. (i–l) Corresponding barcode representations, highlighting the presence of well-defined Na–Na hopping distances and a robust, cavity-rich framework in NTSC, in contrast to the more fragmented topology of NTC.

The topological evolution of the non-Na framework is even more pronounced. Quantitative H_1_ persistence analysis shows that the total H_1_ persistence increases from 13.38 Å in the reference structure to 81.94 Å in NTSC, accompanied by increases in the mean H_1_ persistence (0.104–0.788 Å) and maximum H_1_ persistence (0.368–2.090 Å). These results demonstrate the formation of more persistent looplike topological motifs within the amorphous framework after structural modification. Compared with the less persistent framework topology of the reference structure, the enhanced H_1_ persistence indicates a more heterogeneous and interconnected structural environment, which can provide a greater diversity of connected regions for Na-ion migration. This topology evolution agrees with the experimentally identified mixed-anion coordination environment and the distorted Ta-centered framework, where structural disorder generates a more flexible network for ion transport.

Furthermore, the persistent Betti number profiles reveal that the characteristic length scales of the topological features in NTSC overlap with the Na–Na migration distances obtained from structural analysis and AIMD simulations. Therefore, rather than representing isolated migration channels, the topological descriptors provide quantitative evidence that the amorphous NTSC framework possesses a favorable connectivity environment for Na-ion transport.

Collectively, the combination of AIMD simulations and quantitative topological analysis demonstrates that NTSC achieves enhanced Na^+^ transport through the synergistic effects of shortened Na–Na hopping distances, modified Na coordination environments, and a more persistent framework topology. These features provide a structural explanation for the experimentally observed low activation energy and high ionic conductivity of amorphous NTSC, highlighting the role of mixed-anion amorphization in constructing fast-ion-conducting networks beyond conventional crystalline electrolytes.

To further assess the practical viability of NTSC as a Na-ion solid-state electrolyte, all-solid-state sodium-ion batteries were assembled and evaluated at room temperature. To evaluate its electrochemical stability, linear sweep voltammetry (LSV) was conducted, and the results show that NTSC is electrochemically stable between 2.35 and 3.85 V (vs Na_15_Sn_4_) (Figure S11). A symmetric Na_15_Sn_4_|NTSC|Na_15_Sn_4_ cell was assembled to assess the interfacial stability of the solid electrolytes against the Na_15_Sn_4_ anode. The time-dependent EIS results show that the interfacial resistance increases significantly during 40 h from 418 to 4302 Ω (Figure S10), indicating its instability with a typical anode for Na-based SSBs. The increased resistance might originate from the reduction of Ta^5+^.^26^ Half-cells with the configuration Na_15_Sn_4_|Na_3_PS_4_|NTSC|NaCrO_2_/NTSC/C were employed (Figure 5a). Owing to the intrinsic electrochemical instability of NTSC at low potentials, Na_3_PS_4_, which exhibits an ionic conductivity of 0.21 mS/cm, was introduced to stabilize the interface with the Na_15_Sn_4_ anode (Figure S13).

**Figure 5:**
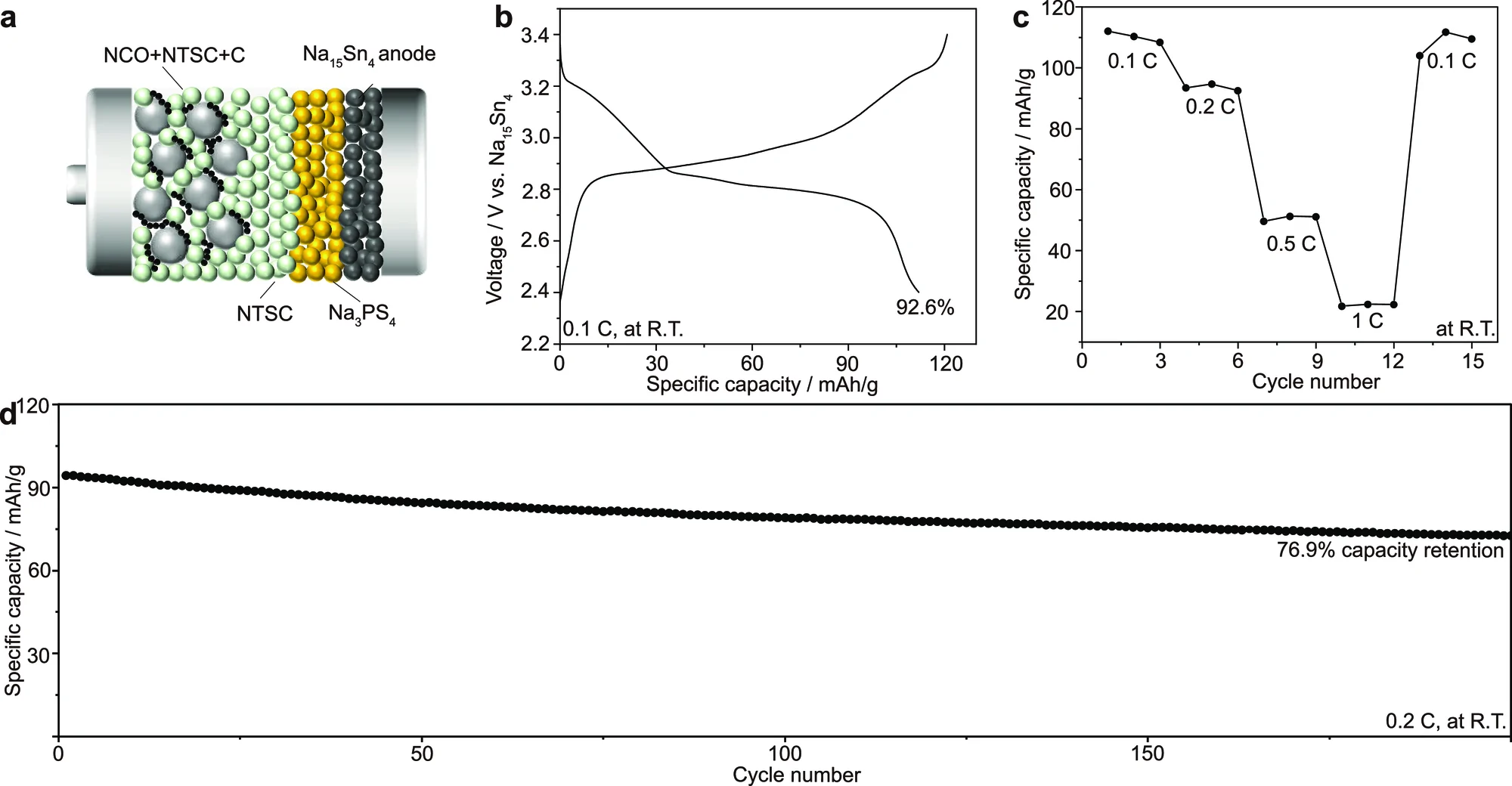
Electrochemical performance of NTSC-based all-solid-state sodium-ion batteries. (a) Schematic illustration of the NTSC-based solid-state battery configuration (Na_15_Sn_4_|Na_3_PS_4_|NTSC|NaCrO_2_/NTSC/C). (b) Initial galvanostatic charge–discharge profiles recorded at 0.1 C and room temperature. (c) Rate performance of the NTSC-based cell at different current densities, demonstrating reversible Na^+^ storage. (d) Long-term cycling performance measured at 0.2 C, showing stable capacity retention over 200 cycles at room temperature.

Figure 5b shows the initial galvanostatic charge–discharge profiles of the NTSC-based cell recorded at 0.1 C within a voltage window of 2.4–3.4 V versus Na_15_Sn_4_. At room temperature, the cell delivers a high initial discharge capacity of 111.95 mAh/g with an initial Coulombic efficiency of 92.6% (Figure 5b), demonstrating good electrochemical reversibility. With increasing current density, reversible discharge capacities of 93.48, 51.25, and 21.78 mAh/g are achieved at 0.2, 0.5, and 1 C, respectively (Figure 5c). The noticeable capacity decay at higher C-rates, particularly beyond 0.5 C, is primarily attributed to the limited ionic conductivity of Na_3_PS_4_.^13^ Long-term cycling performance was further evaluated at 0.2 C. As shown in Figure 5d, the cell exhibits excellent cycling stability, retaining 76.9% of its initial discharge capacity after 200 cycles with stable Coulombic efficiency. This sustained performance highlights the favorable electrochemical compatibility of NTSC with the NaCrO_2_ cathode.

Overall, these results demonstrate that NTSC enables highly reversible Na^+^ storage and robust long-term cycling stability in all-solid-state sodium-ion batteries at room temperature.

## Conclusion

We report a new amorphous superionic Na-ion solid-state electrolyte, TaCl_5_-0.5Na_2_S (NTSC), synthesized via high-energy ball-milling from TaCl_5_ and Na_2_S precursors. Owing to its mixed-anion amorphous framework, NTSC exhibits a high room-temperature ionic conductivity of 2.9 mS cm^–1^. When implemented in all-solid-state sodium-ion batteries with the configuration Na_3_Sn_4_|Na_3_PS_4_|NTSC|NaCrO_2_/NTSC/C, the electrolyte enables a high initial discharge capacity of 111.95 mAh/g, a Coulombic efficiency of 92.6%, and excellent cycling stability, retaining 76.9% of its capacity after 200 cycles at 0.2 C.

Comprehensive structural characterization reveals that NTSC consists of Ta-centered bipolyhedra interconnected via corner-sharing sulfur atoms [Ta_2_Cl_10_S], [TaCl_5_S], [TaCl_4_S], and [TaCl_4_S_2_], forming a highly disordered yet structurally coherent amorphous network. This framework weakens Na^+^-anion interactions and creates a flexible, disordered environment that supports fast Na^+^ migration. Consistent with these experimental observations, ab initio molecular dynamics simulations identify continuous Na^+^ diffusion pathways and a low migration barrier, confirming the intrinsic superionic nature of NTSC. Further topological analysis demonstrates that sulfur-induced lattice distortions shorten Na–Na hopping distances and generate persistent, multidimensional transport cavities, providing a topology-level explanation for the experimentally observed low activation energy and high ionic conductivity.

Together, these results highlight how mixed-anion chemistry and amorphization can be synergistically exploited to overcome the transport limitations of conventional halide electrolytes. This work not only introduces a new class of high-performance amorphous Na-SSEs but also establishes clear structural and topological design principles for engineering next-generation solid electrolytes with enhanced ionic conductivity and improved electrochemical compatibility.

## Experimental Section

### Materials Synthesis

#### NTSC Solid-State Electrolytes

Na_2_S (99.5%, Sigma-Aldrich) and TaCl_5_ (99.8%, Sigma-Aldrich) were weighed to the stoichiometric molar ratio and mechanically mixed in a ZrO_2_ container with ZrO_2_ balls (diameter = 5 and 10 mm, 1:1, wt %) in a planetary ball mill (Fritsch, Pulverisette 7) at 500 rpm for 40 h. The powder obtained was collected for further use.

#### Na_3_PS_4_ (NPS) Solid-State Electrolytes

NPS was synthesized by ball-milling a stoichiometric mixture of Na_2_S and P_2_S_5_ in a ZrO_2_ container with ZrO_2_ balls (diameter = 10 mm, ball to powder weight ratio = 30:1) in a planetary ball mill (Retsch PM100) at 550 rpm for 3 h. The obtained powder was pelletized (at 150 MPa) and further annealed at 270 °C for 2 h.

#### Na–Sn Alloy

Na and Sn with a stoichiometric molar ratio (15:4) were added to ball-milling jars in a glovebox and further mechanically mixed for 4 h at 500 rpm. The obtained powder was collected for further use.

#### Preparation of Cathode Composites

The cathode composite was obtained through mixing NaCrO_2_, NTSC, and C together with a weight ratio of 65:30:5 at 150 rpm for 2 h.

### Characterizations

#### XRD

The X-ray diffraction (XRD) measurements were performed on a Bruker Davinci 1 with Cu Kα radiation (λ = 1.54178 Å) at room temperature. The data were collected by scanning 1s per step with a step width of 0.02 from 10 to 80° (2θ). High-resolution X-ray data were refined using the Rietveld method (Bruker TOPAS V6 software). The background was modeled with a Chebyshev polynomial function of fifth order, and the fundamental parameter approach was chosen as the peak shape function.

#### SEM

The morphologies of solid electrolytes and cathode composites were characterized by an FEI Apreo emission scanning electron microscope (SEM) equipped with energy-dispersive spectroscopy (EDS).

#### DSC

Approximately 10 mg of the sample was sealed in an aluminum pan and heated under continuous nitrogen flow to prevent degradation. Measurements were conducted from 25 to 500 °C at a heating rate of 10 °C/min.

#### Raman

Raman tests were conducted by a Renishaw RM-1000 spectrometer with a laser wavelength of 532 nm.

#### XPS

Chemical characterization of the samples and the state of the surface were obtained with X-ray Photoemission Spectroscopy (XPS). Core-level spectra were measured using a monochromatized Al Kα (1486.6 eV) line with a hemispherical analyzer at NTNU XPS facilities and a Mg K*α* (1253.6 eV) nonmonochromatized light. Measurements were performed at room temperature and with chamber pressures below 10^–9^ mbar. The calibration of the binding energy was done using C 1s and Au 4f reference peaks. Peak fitting was optimized using CasaXPS software.

#### XAS

Sulfur and chlorine K-edge XAS spectra were collected at the bending magnet ASTRA beamline at the SOLARIS National Synchrotron Radiation Center (Krakow, Poland). The measurements were performed in transmission and fluorescence modes at room temperature using an incident photon beam provided by a modified Lemonnier-type double-crystal monochromator equipped with Si(111) crystals. Energy calibration at the S K-edge was carried out using ZnSO_4_·7H_2_O (white-line position at 2481.4 eV), while calibration at the Cl K-edge was performed using KCl (white-line position at 2825.0 eV). The measurements were conducted using ionization chambers, with the ionization and sample chambers filled with pure N_2_ at pressures of 25 and 27 Torr, respectively. For the measurements, the powder samples were sealed between two sulfur-free and chlorine-free tapes in a glovebox under an argon atmosphere, forming a thin, uniform layer. The final spectra were obtained by merging at least three consecutive scans.^27^ The sodium K-edge XAS spectra were collected in fluorescence and total electron yield (TEY) modes at the PIRX beamline at the SOLARIS.^28^

The energy resolution was at the level of 200 meV. Tantalum L_3_-edge spectra were collected at the BM31 beamline in ESRF with sealing BN-NTSC or BN-TaCl_5_ mixture (80:20, wt %) (pellet shape) within an aluminum laminate film. All spectra were processed by using the Athena program from the Demeter software package.

#### xPDF

xPDF tests for TaCl_5_, NTC, and NTSC were conducted at the BM31 beamline in ESRF with sealed samples in a capillary.

### Electrochemical Measurements

### Impedance Spectroscopy

The Na-ion conductivity of solid electrolytes was measured by adding 0.15 g of solid electrolyte powder into a PEEK (polyether ether ketone) cell with a diameter of 10 mm and further pressed at 300 MPa. EIS measurements were carried out at 375 MPa and room temperature (25 °C, unless specified) in the frequency range of 10–7 MHz with 10 mV of applied sinus amplitude on a VMP-300 potentiostat (BioLogic). The collected impedance data were analyzed and fitted using EC-LAB software based on the method of Randomize+simplex. To determine the individual contributions of the bulk (*R*_b_) and grain boundary (*R*_gb_) resistances, the Nyquist plots were fitted to an equivalent circuit model consisting of *R*_1_ + *Q*_2_/*R*_2_.

#### Half-Cell Assembling

40 mg of NPS was added to the PEEK cell with a diameter of 10 mm and pressed at 375 MPa; then, 40 mg NTSC powder was placed on one side of the NPS pellet and pressed at the same pressure; the cathode composite was placed on top of NTSC and pressed at 375 MPa; finally, 80 mg of Na–Sn alloy was homogeneously added on the other side of the NPS pellet and pressed at 200 MPa. The half-cells were tested at room temperature (25 °C) at 120 MPa pressure using a Neware battery cycler.

#### Linear Sweep Voltammetry (LSV)

The LSV test was conducted with a scan rate of 0.05 mV/s with a cell configuration of NTSC + CB|NTSC|NPS|Na_15_Sn_4_.

### Computational Methods

#### Density Functional Theory Calculations (DFT) and Structural Optimization

All first-principles calculations were performed within the framework of DFT using the Vienna *Ab initio* Simulation Package (VASP).^29^ The interaction between core and valence electrons was described by the projector augmented wave (PAW) method, and the exchange-correlation functional was treated using the Perdew–Burke–Ernzerhof (PBE) generalized gradient approximation.^30^

To obtain highly accurate geometries and reliable bond lengths for subsequent dynamical and topological analyses, all structures were fully optimized using a high-precision setup. The plane-wave kinetic energy cutoff was set to 520 eV. The electronic self-consistent convergence criterion was 10^–5^ eV, and ionic relaxations were stopped when the residual forces on each atom were smaller than 0.005 eV/Å.

Geometry optimizations were performed using the conjugate-gradient algorithm. A 3 × 3 × 1 Monkhorst–Pack *k*-point mesh was employed for Brillouin zone sampling. The real-space projection approximation was disabled (LREAL = .FALSE.) and a finer FFT grid was used (ADDGRID = .TRUE.) to ensure numerical accuracy. Nonspherical contributions inside the PAW spheres were explicitly included (LASPH = .TRUE.), which is essential for systems containing the heavy Ta element. Symmetry was switched off (ISYM = 0) to avoid artificial constraints on local structural distortions induced by S substitution.

#### Ab Initio Molecular Dynamics Simulations

An amorphous NTSC structural model was generated using a three-stage melt–quench ab initio molecular dynamics procedure.^20^ The initial crystalline structure was first heated from 300 to 1500 K over 5 ps using a 2 fs time step, followed by a 5 ps equilibration at 1500 K to allow sufficient structural relaxation in the molten state. The equilibrated melt was subsequently quenched from 1500 to 300 K over 6 ps, corresponding to an average cooling rate of approximately 200 K ps^–1^. The total melt–quench simulation time was 16 ps, comprising 8000 MD steps. All melt–quench simulations were performed within the NVT ensemble using a Nosé–Hoover thermostat with a fixed simulation cell. The electronic structure calculations employed a plane-wave cutoff energy of 350 eV and an electronic convergence criterion of 10^–5^ eV. The Γ-point approximation was used, crystal symmetry was disabled during the molecular dynamics simulations, and Gaussian smearing with a width of 0.05 eV was adopted. The final configuration obtained after quenching to 300 K was used as the amorphous structural model for subsequent structural and ion-transport analyses.

The resulting amorphous Na_36_Ta_36_S_18_Cl_180_ structure was subsequently subjected to extended AIMD simulations at 600, 800, 1000, and 1200 K to characterize the temperature-dependent Na^+^ transport behavior. All simulations were performed in the NVT ensemble using a Nosé–Hoover thermostat with a fixed simulation cell and a time step of 1 fs. Each trajectory contained 30,000 MD steps, corresponding to a total simulation time of 30 ps. The plane-wave cutoff energy was set to 480 eV, and the electronic self-consistent-field convergence criterion was set to 10^–5^ eV. Gaussian smearing with a width of 0.05 eV was employed, while crystal symmetry was disabled throughout the simulations. The projector treatment employed the automatic real-space projection scheme, with nonspherical gradient corrections and the additional support grid enabled. The resulting trajectories were used to calculate the Na^+^ mean-squared displacement and diffusion coefficients.

#### Diffusion Coefficient and Ionic Conductivity

Na^+^ mean square displacements (MSDs) were calculated from the AIMD trajectories, and diffusion coefficients were obtained using the Einstein relation^31^

$$D=\lim_{t\to \infty }\frac{1}{6t}\left\langle {\left|r\left(t\right)-r\left(0\right)\right|}^{2}\right\rangle$$

where *r*(*t*) denotes the position of a Na^+^ ion at time *t*.

The temperature dependence of the diffusion coefficients was fitted using the Arrhenius equation

$$D\left(T\right)=D_{0}\exp\left(-\frac{E_{a}}{k_{B}T}\right)$$

The ionic conductivity was estimated using the Nernst–Einstein relation^22^

$$\sigma =\frac{nq^{2}D}{k_{B}T}$$

where *n* is the number density of mobile Na ions, *q* is the elementary charge, *k*_B_ is the Boltzmann constant, and *T* is the temperature. The room-temperature ionic conductivity was obtained by extrapolating the Arrhenius fit to 300 K.

#### Radial Distribution Function (RDF) Analysis

Radial distribution functions (RDFs) between Na–S and Na–Cl pairs were computed from the AIMD trajectories by time averaging over the production runs to characterize the local chemical environment of Na ions in TaCl_5_-0.5Na_2_S and NaTaCl_6_.

#### Topological Data Analysis and Persistent Homology

Persistent homology, a topological data analysis (TDA) approach, provides a rigorous framework for quantifying multiscale topological features in complex geometric data.^24,25^ In TDA, atomic configurations are represented using simplicial complexes composed of simplices, where a *k*-simplex is defined by *k* + 1 vertices (e.g., points, edges, triangles, and tetrahedra for *k* = 0–3). A filtration is constructed by generating a sequence of nested simplicial complexes with an increasing length scale parameter ε

$$Ø\subseteq K\left(\epsilon_{1}\right)\subseteq K\left(\epsilon_{2}\right)...\subseteq K\left(\epsilon_{n}\right)$$

At each scale, topological features such as connected components, loops, and cavities are quantified using homology. The number of independent *k*-dimensional features is given by the Betti number β_*k*_. Persistent homology tracks the emergence (birth) and disappearance (death) of these features along the filtration. Features with long lifetimes are considered topologically robust, whereas short-lived features are typically associated with noise. The resulting multiscale information is visualized using persistence barcodes or persistence diagrams.

To probe topological characteristics relevant to Na^+^ transport, persistent homology analysis was performed on two-point clouds derived from each structure:^32^ (i) the Na^+^ sublattice and (ii) the non-Na framework comprising the Ta–Cl/S network. For a point set $X=\{x_{i}\}\subset R^{3}$ , Vietoris–Rips (VR) complexes VR **(*X,r*) were constructed by connecting points separated by distances smaller than a filtration parameter *r*, with higher-dimensional simplices added when all lower-dimensional faces were present. Increasing *r* generated a nested filtration

$$\mathrm{VR}\left(X,r_{1}\right)\subseteq \mathrm{VR}\left(X,r_{2}\right)\subseteq \cdot \cdot \cdot \subseteq \mathrm{VR}\left(X,r_{n}\right),r_{1}<r_{2}<\cdot \cdot \cdot <r_{n}$$

At each filtration scale, Betti numbers were computed, where β_0_ represents the number of connected components and β_1_ corresponds to independent looplike features. The 0D persistence (β_0_) of the Na^+^ sublattice was used to extract characteristic Na–Na connectivity and hopping length scales, as the death times reflect distances at which isolated Na sites become connected. In contrast, the 1D persistence (β_1_) of the non-Na framework was analyzed to quantify the presence and stability of looplike cavities that can accommodate and facilitate Na^+^ migration. All persistent homology calculations were carried out using the Ripser package.^33^

#### Na-Ion Migration Pathways

Na^+^ migration pathways were visualized by accumulating Na^+^ trajectories from AIMD simulations and projecting them onto the crystallographic planes. Regions with high probability densities were rendered to identify continuous Na^+^ diffusion channels.

## Supplementary Material





## Notes

Optical images; X-ray diffraction patterns; scanning electron microscopy images; differential scanning calorimetry curves; temperature-dependent electrochemical impedance spectra; Na K-edge and Cl K-edge XANES spectra; amorphous structural model and temperature-dependent Na^+^ diffusion behavior obtained from AIMD simulations; mean-squared displacement analysis; LSV measurements; SEM images and EDS elemental mapping of cathode composites; room-temperature electrochemical impedance spectrum of the Na_3_PS_4_ electrolyte; cross-sectional SEM images of cycled cathode composites; and comparison of ionic conductivity and *E*_a_ of amorphous chloride-based sodium-ion conductors (PDF)
VASP density functional theory data (POSCAR) (ZIP)
