# Supplementary material for: Sulfur-Induced Lattice Disordering in Halide Electrolytes Enables Superionic Sodium-Ion Conduction
Source: ACS Nano. 2026 Oct 29;20(44):29709–19. doi: 10.1021/acsnano.6c11189 (PMC13758389; doi:10.1021/acsnano.6c11189)
Supplement: Supplementary file 1 [file nn6c11189_si_001.pdf]

Supplemental information

Sulfur-Induced Lattice Disordering in Halide Electrolytes Enables Superionic Sodium Ion Conduction

Feng Jin[1,2†], Bingxu Wang[1†], Meenal Agrawal[2], Ingeborg Sellæg Ellingsen[2], Guobiao Jin[1], Lulu Alluhaibi[3], Alexey Maximenko[3], Marcin Zając[3], Dragos Stoian[4], Kenneth Marshall[4], Wouter Van Beek[4], Feng Pan[1], Daniel Rettenwander[2,5-7**]*

[1]School of Advanced Materials, Peking University Shenzhen Graduate School, Shenzhen, 518055, China

[2]Department of Material Science and Engineering, NTNU Norwegian University of Science and Technology, Trondheim 7491, Norway

[3]National Synchrotron Radiation Centre SOLARIS, Jagiellonian University, Czerwone Maki 98, 30-392, Kraków, Poland

[4]Swiss-Norwegian Beamlines, European Synchrotron Radiation Facility, 71 Ave. des Martyrs, Grenoble 38000, France

[5]Christian Doppler Laboratory for Solid-State Batteries, NTNU Norwegian University of Science and Technology, Trondheim 7491, Norway

[6]AIT Austrian Institute of Technology GmbH, Giefinggasse 2, Vienna, 1210, Austria.

[7]Institute of Chemical Technologies and Analytics, Vienna University of Technology, Getreidemarkt 9, Vienna, 1060, Austria.

[†]Feng Jin and Bingxu Wang contributed equally to this work.

Corresponding authors: [*]panfeng@pkusz.edu.cn, [**]daniel.rettenwander@ntnu.no

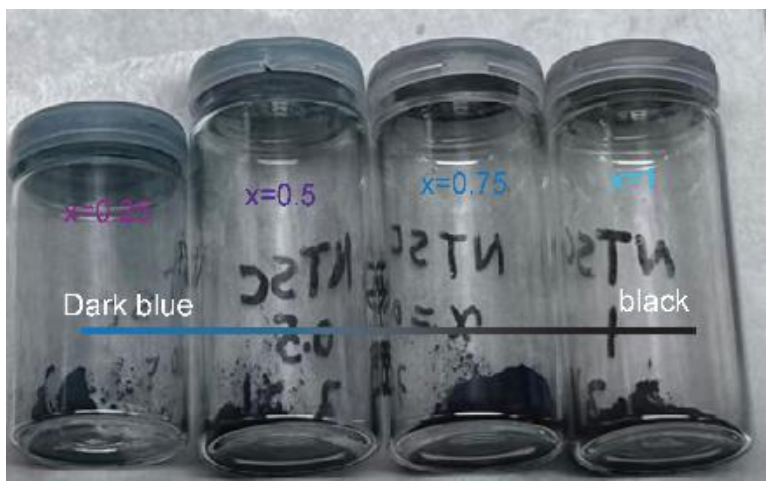

Figure S1: Optical images of $TaCl_5 \cdot xNa_2S$ (x=0.25, 0.5, 0.75, 1)

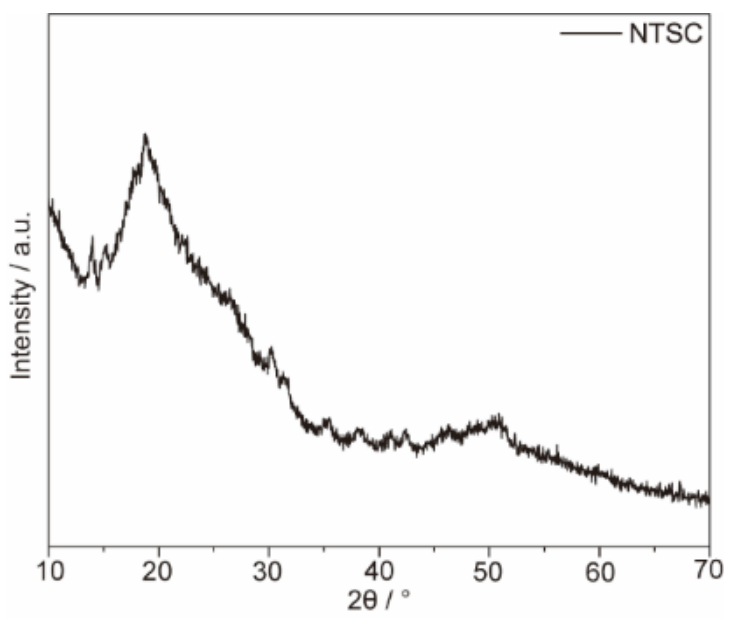

Figure S2: XRD pattern of NTSC.

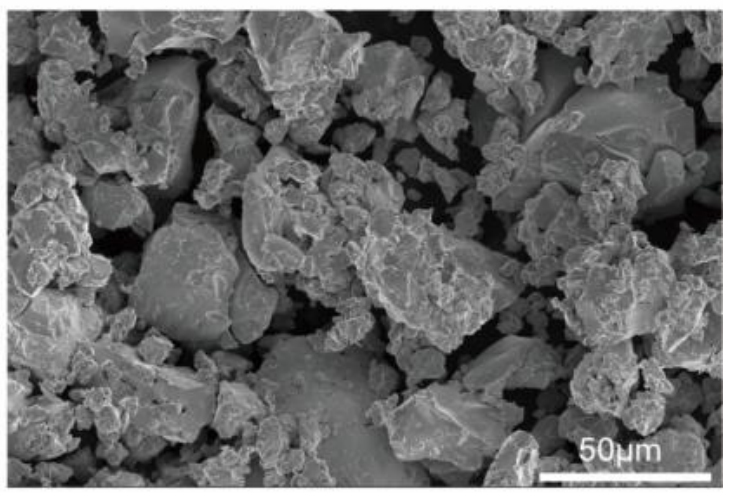

Figure S3: SEM image of NTSC.

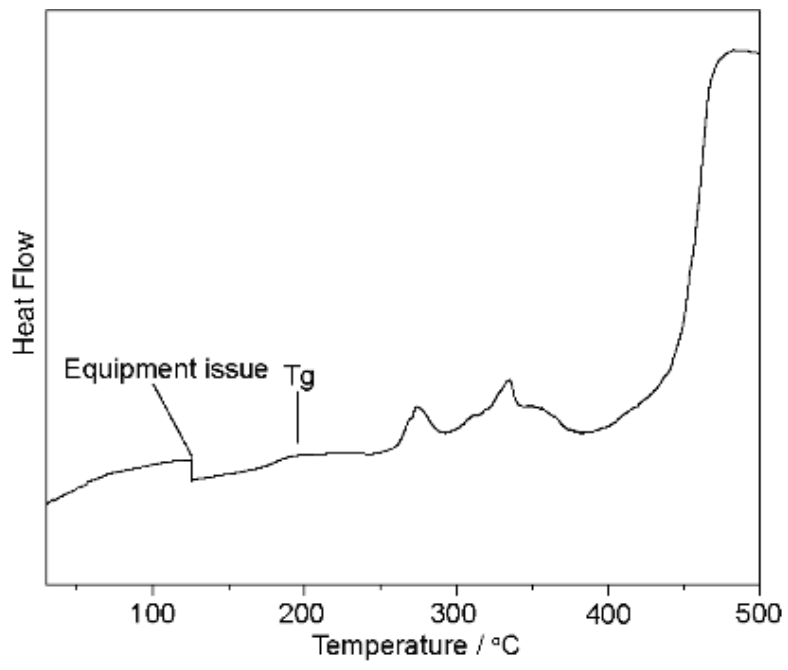

Figure S4. DSC curves of NTSC.

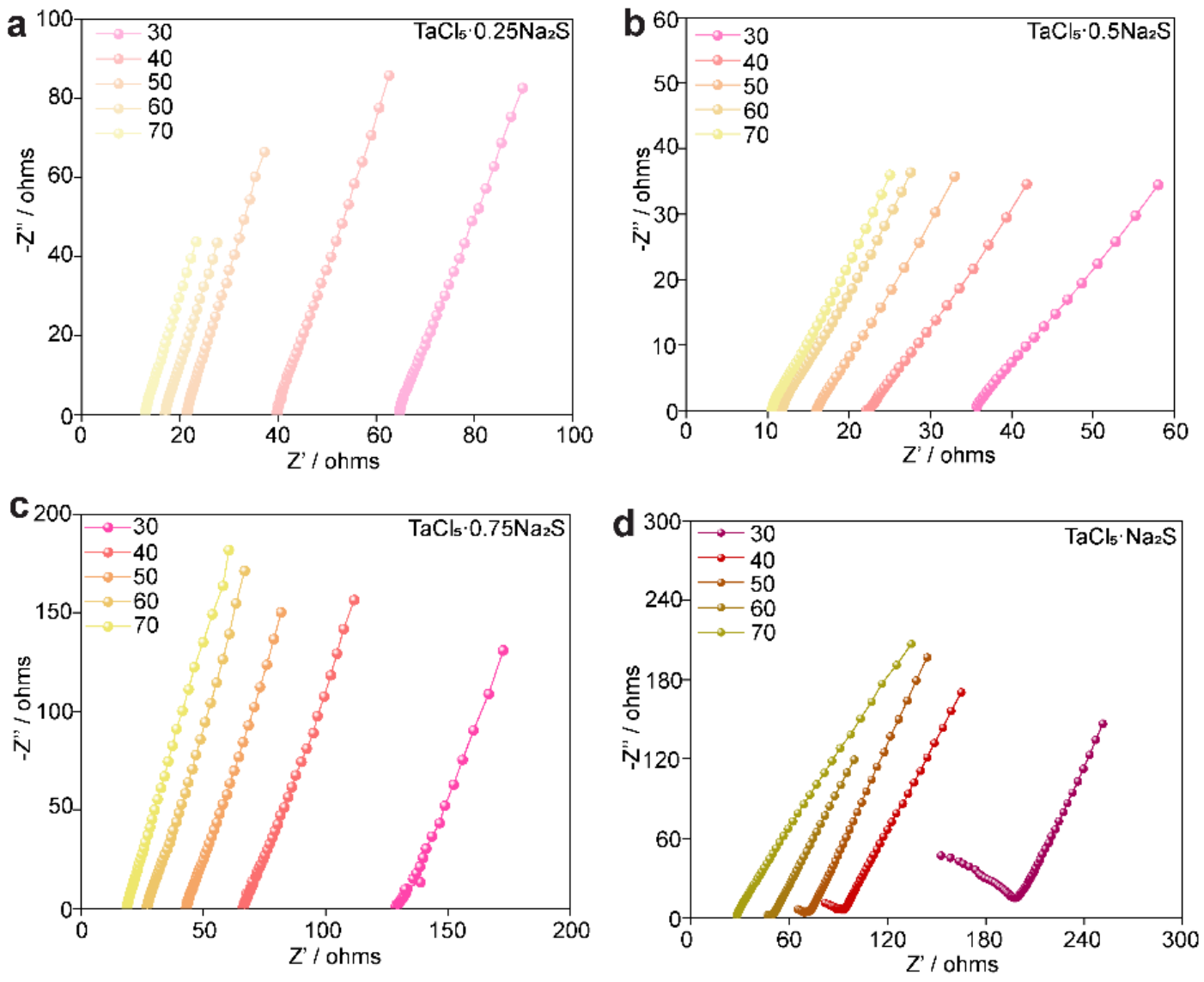

Figure S5: The Nyquist plots of $TaCl_5{\cdot}xNa_2S$ at various temperatures.

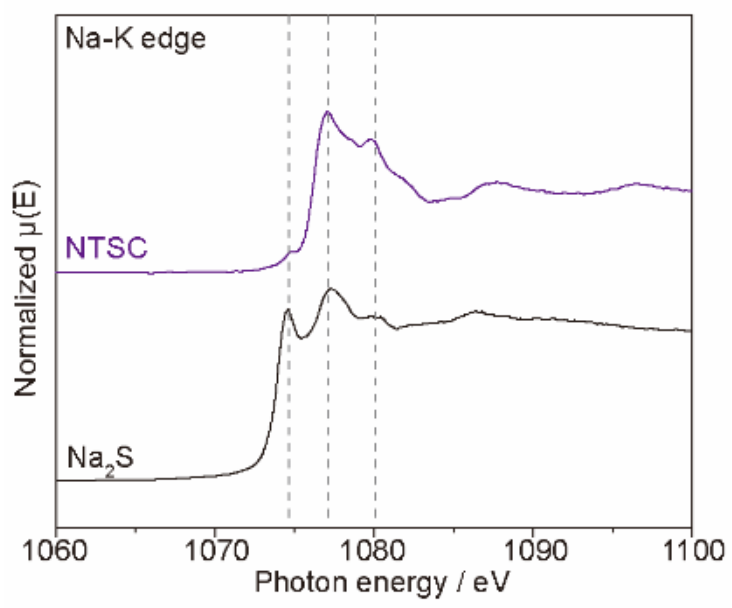

Figure S6. Na *K-edge* XANES spectra of Na_2S precursor and NTSC.

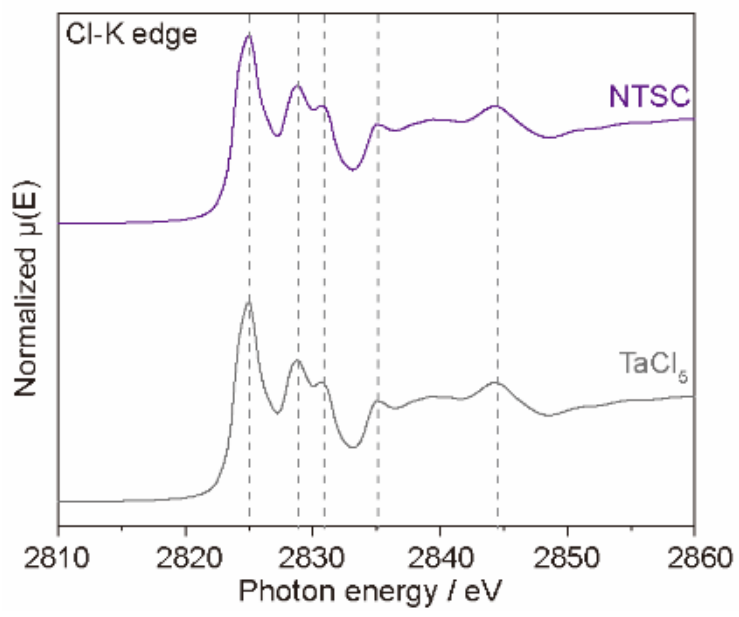

Figure S7. Cl *K-edge* XANES spectra of $TaCl_5$ precursor and NTSC.

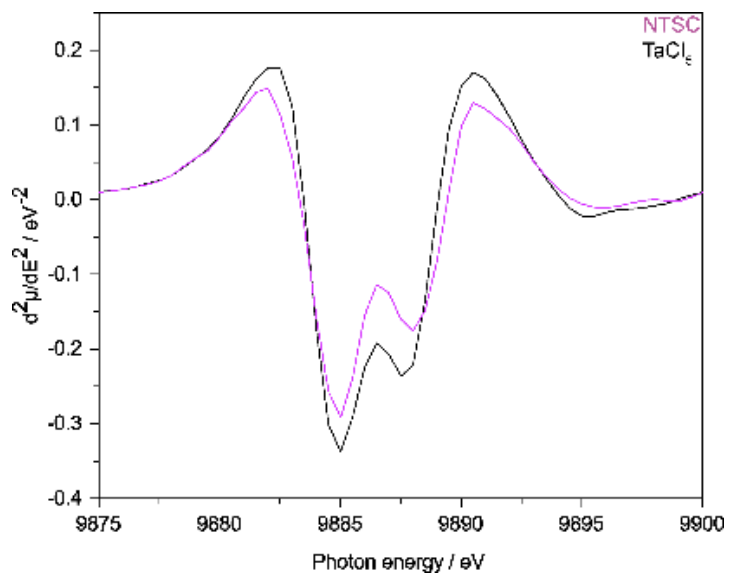

Figure S8. Second derivative XANES spectra of $TaCl_5$ and NTSC.

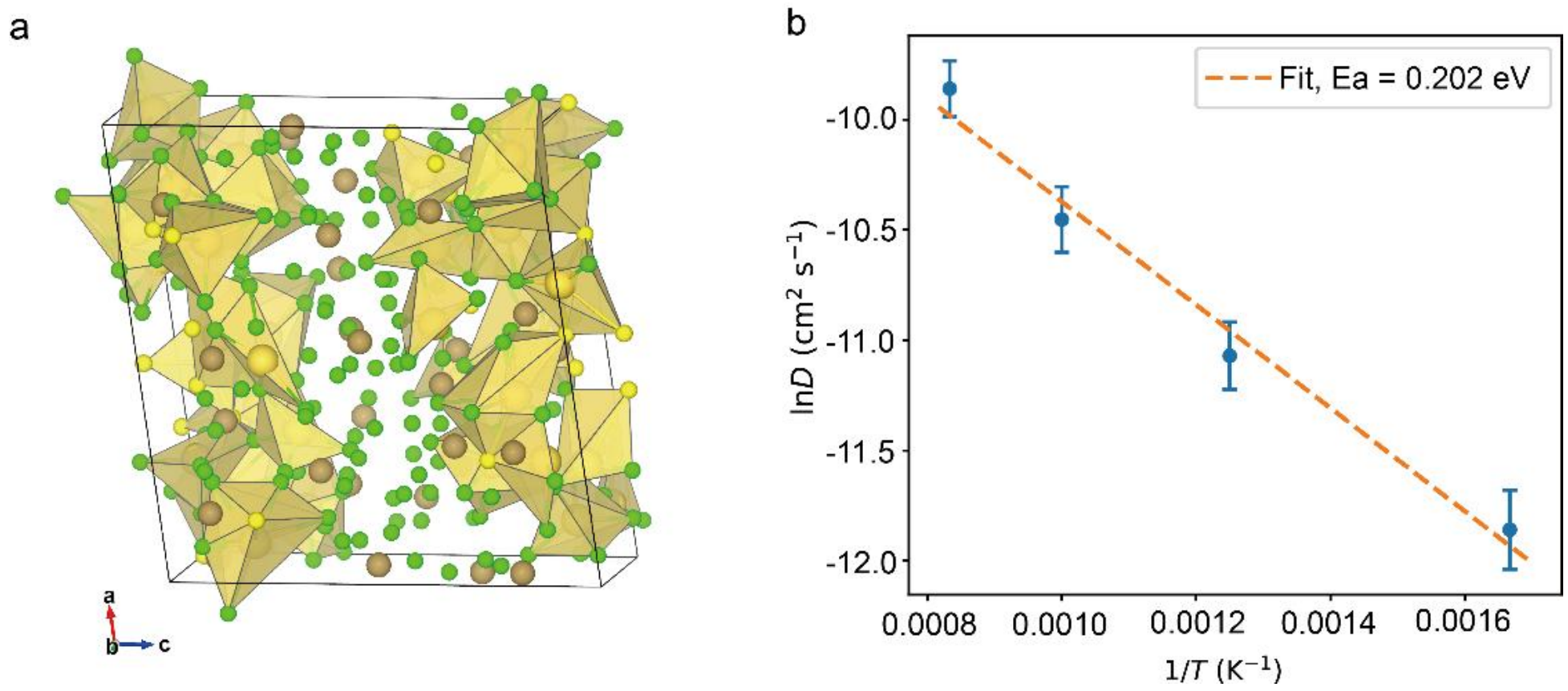

Figure S9. Amorphous structural model and temperature-dependent Na^+ diffusion behavior of NTSC. (a) Amorphous NTSC structure generated using a melt–quench ab initio molecular dynamics procedure. The structure exhibits pronounced spatial heterogeneity, with Na/S/Cl-rich regions distributed within a Ta-rich framework. The interconnected Na-rich regions provide structurally accessible environments that may facilitate Na^+ migration. (b) Arrhenius relationship of the Na^+ diffusion coefficients obtained from 30 ps AIMD simulations at 600, 800, 1000, and 1200 K. Error bars represent the estimated uncertainties obtained from independent trajectory intervals. The fitted Arrhenius relationship gives an apparent activation energy of 0.202 eV, and extrapolation to 300 K yields a diffusion coefficient of 1.33×10^{-7} $cm^2\ s^{-1}$ and a Nernst-Einstein ionic conductivity of 3.18 mS cm^{-1}.

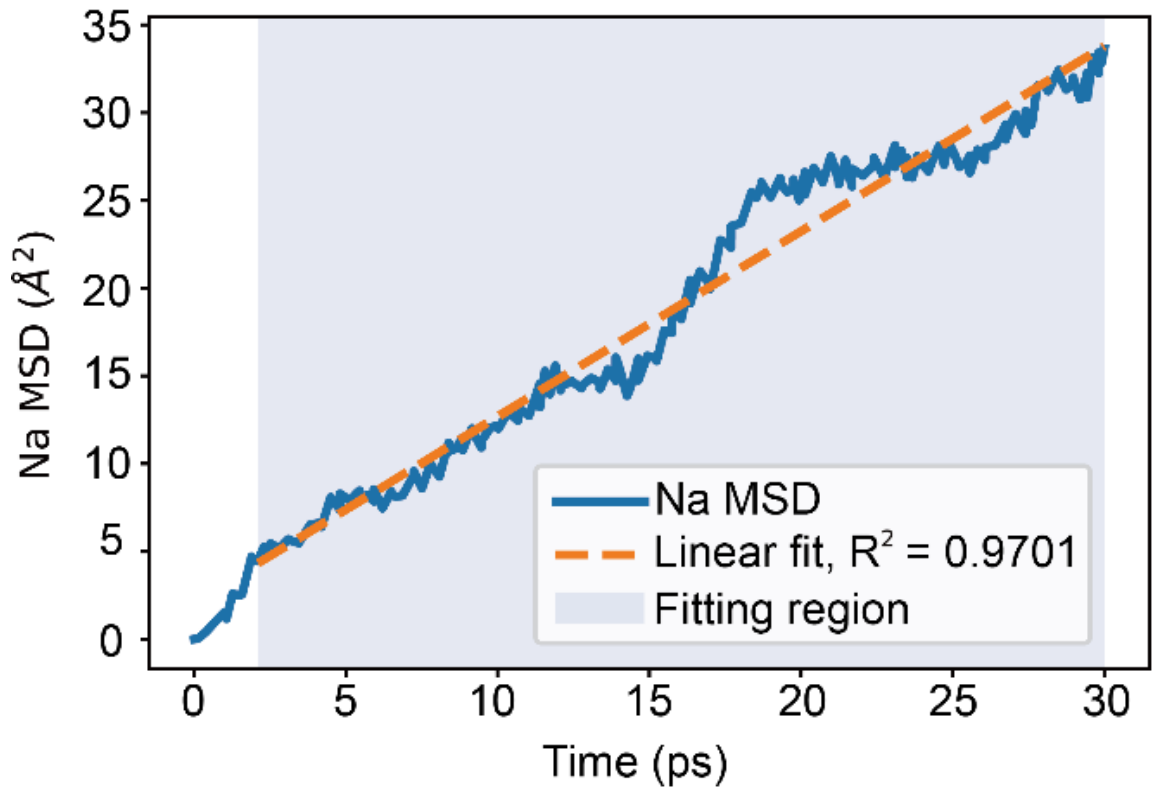

Figure S10**.** Mean-squared displacement of Na^+ ions in amorphous NTSC obtained from AIMD simulation at 800 K. The initial approximately 2 ps transient regime was excluded from the diffusion analysis, and the diffusion coefficient was determined from the subsequent linear diffusive regime. The linear fit gives $R^2 = 0.9701$, indicating a well-defined diffusive regime for Na^+ transport.

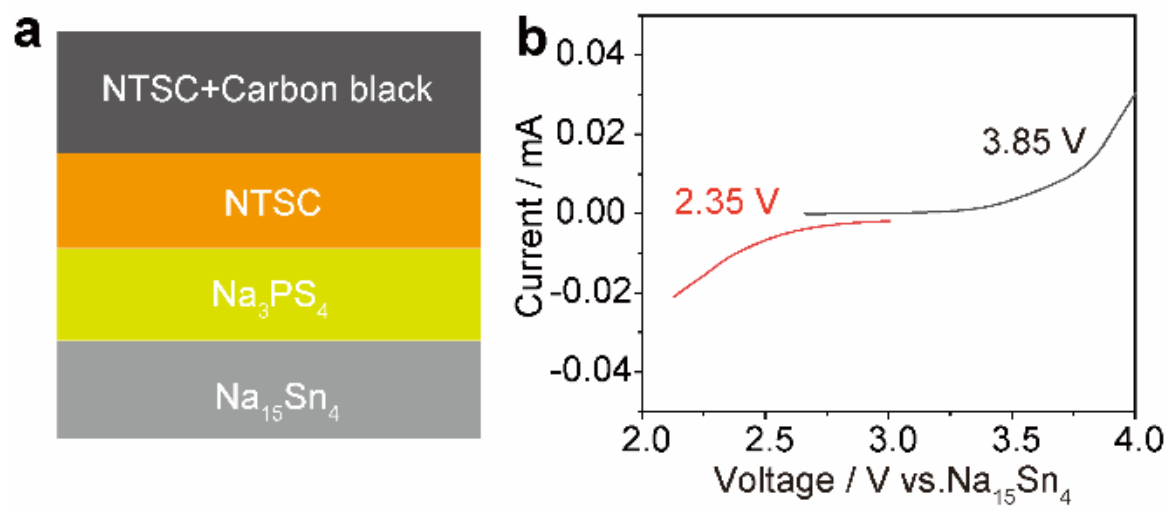

Figure S11. (a)Schematic illustration of the cell used for LSV test, containing NTSC & Carbon black as cathode, NTSC & NPS as electrolytes and $Na_{15}Sn_4$ as anode, respectively. (b) LSV curve of the $0.5Na_2S$-$TaCl_5$.

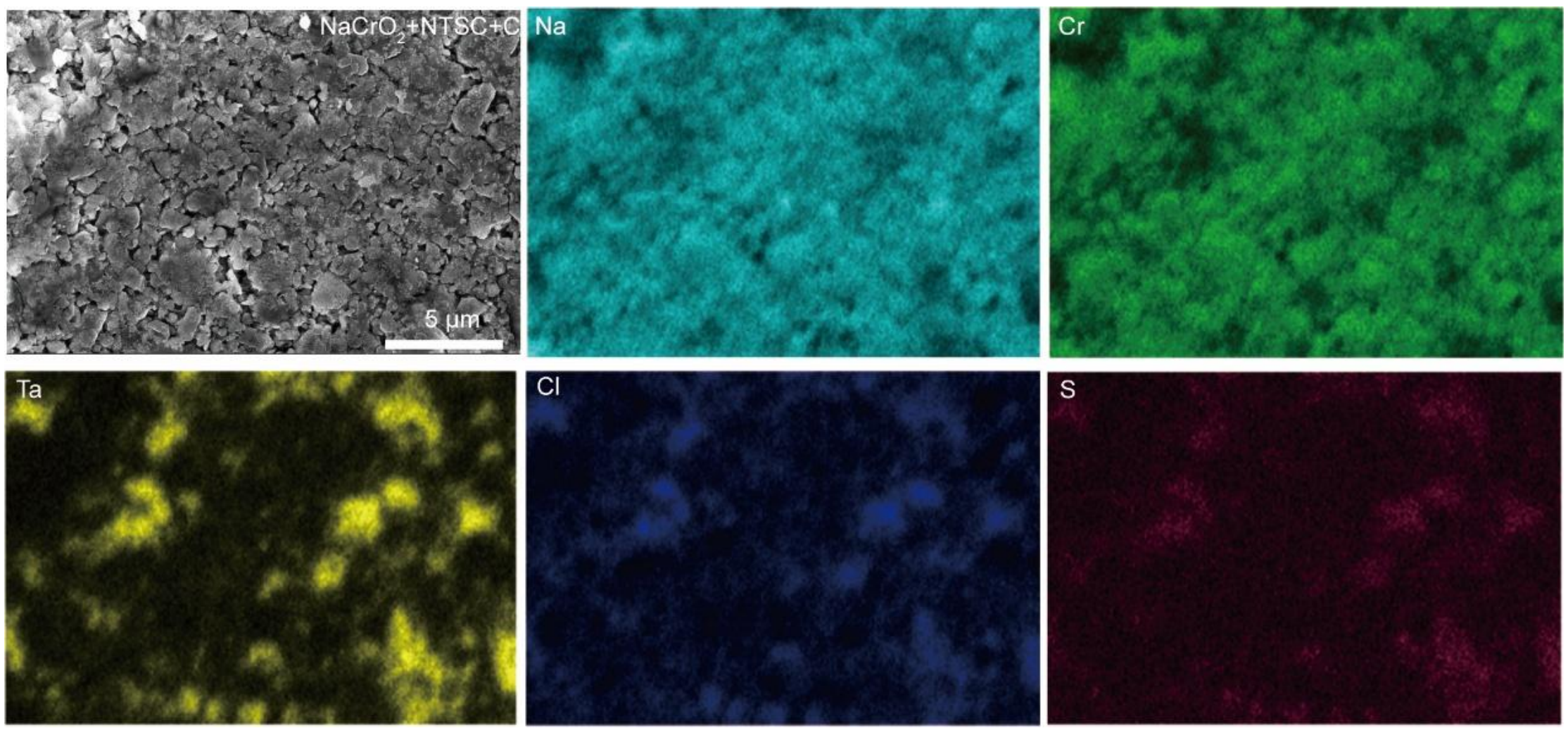

Figure S12. SEM images and corresponding EDS mapping of cathode composites, containing $NaCrO_x$, NTSC and C.

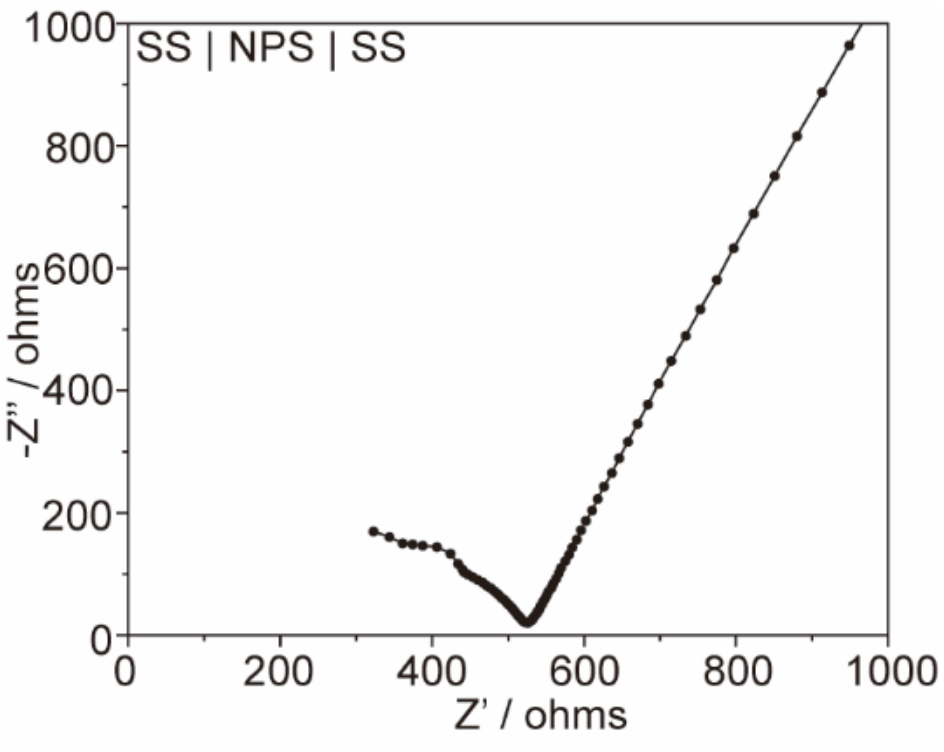

Figure S13. Nyquist plot of NPS SE at room temperature (25 ºC). The EIS test was performed based on SS | NPS | SS (stainless steel) cell in PEEK cell with a diameter of 10 mm at room temperature and NPS exhibits ionic conductivity of ~ 0.21 mS/cm.

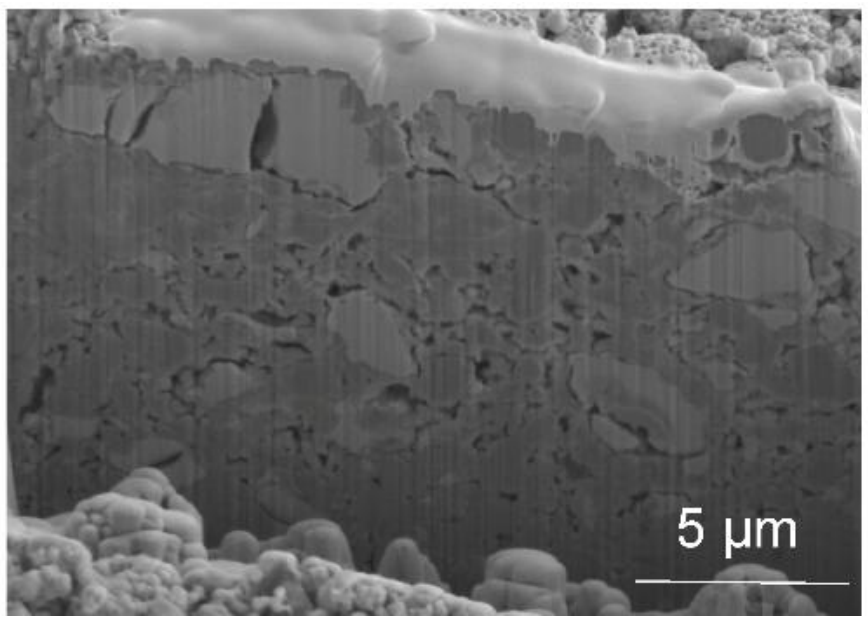

Figure S14. Cross-section SEM images for cycled cathode composites.

Table 1. Summary of the ionic conductivity of amorphous chloride Na-ion conductors

Na-SSE	Ionic conductivity mS/cm	Activation energy (Ea / eV)	References
$NaTaCl_6$	4	0.30	[1]
$0.5Na_2O_2$-$TaCl_5$	4.62	0.30	[2]
2NaF-$ZrCl_4$	0.23	0.28	[3]
Na_2S-3.3$ZrCl_4$	0.498	0.351	[4]
$0.5Na_2CO_3$-$TaCl_5$	5.01	0.355	[5]
Na_2S-1.3$HfCl_4$	0.452	/	[6]
$0.5Na_2S$-$TaCl_5$	2.9	0.276	This work

References:

[1] Y. Hu, J. Fu, X. Lin, J. Xu, J. Luo, F. Zhao, Y. Liu, W. Li, J. T. Kim, H. Su, X. Hao, H. Ren, M. Yang, Y. Huang, X. Sun, "Superionic Amorphous $NaTaCl_6$ Halide Electrolyte for Highly Reversible All- Solid-State Na-ion Batteries" *Matter* **2024**, *7*, 1018–1034.

[2] X. Lin, Y. Zhao, C. Wang, J. Luo, J. Fu, B. Xiao, Y. Gao, W. Li, S. Zhang, J. Xu, F. Yang, X. Hao, H. Duan, Y. Sun, J. Guo, Y. Huang, X. Sun, "A Dual Anion Chemistry-Based Superionic Glass Enabling Long-Cycling All-Solid-State Sodium-Ion Batteries" *Angew. Chem. Int. Ed.* **2024**, *63*, e202314181.

[3] L. Zhou, S. Zhang, W. Li, B. Li, N. S. Grundish, P. Ren, X. Wang, N. Wu, W. Zhou, Y. Li, "Amorphous-Nanocrystalline Fluorinated Halide Electrolytes with High Ionic Conductivity and High-Voltage Stability" *J. Am. Chem. Soc.* **2025**, *147*, 15136–15145.

[4] Z. L. Dong, Y. Gan, V. Martins, X. Wang, B. Fu, E. Jin, Y. Gao, Y. Hu, X. Lin, Y. Yuan, C. Turner, X. Pang, H. Abdolvand, Y. Huang, T. Sham, Y. Zhao, "Novel Sulfide-Chloride Solid-State Electrolytes with Tunable Anion Ratio for Highly Stable Solid-State Sodium-Ion Batteries" *Adv. Mater.* **2025**, *37*, 2503107.

[5] X. Wang, S. Zhang, J. Yue, X. Wang, Y. Xu, Y. Gong, L. Zhou, C. Zhao, J. Liang, X. Zhu, H. Wu, X. Yan, B. Xiao, M. Li, C. Li, S. Wang, X. Sun, X. Li, "A new class of carbonate–oxychloride solid electrolytes for high-performance sodium-ion all-solid-state batteries" *Energy Environ. Sci.* **2025**, *18*, 9927–9938.

[6] Z. L. Dong, B. Sourav, Y. Gan, V. Martins, X. Wang, A. Mozafarighoraba, R. Zhang, C. Turner, X. Pang, H. Abdolvand, Y. Huang, P. Kaghazchi, T. Sham, Y. Zhao, "Design of Sodium Chalcohalide Solid Electrolytes with Mixed Anions for All-Solid-State Sodium-Ion Batteries" *Adv. Funct. Mater.* **2026**, *36*, e16657.
